# Mediators of the Association Between Stroke and Blood Cells: A Two‐Step Multivariable Mendelian Randomization Study

**DOI:** 10.1002/cns.70903

**Published:** 2026-09-11

**Authors:** Tao Chen, Yan Li, Long Cai, Yijun Liu, Pengchen Kuang, Liuyi Zou, Ping Li

**Affiliations:** ^1^ Department of Critical Medicine Yichun People's Hospital Yichun Jiangxi China; ^2^ Department of Operating Room Yichun People's Hospital Yichun China; ^3^ Department of Endocrinology Yichun People's Hospital Yichun China; ^4^ Department of Respiratory and Critical Care Medicine, The First Affiliated Hospital, Jiangxi Medical College Nanchang University Nanchang Jiangxi China; ^5^ Jiang Xi Hospital of China‐Japan Friendship Hospital Nanchang China

**Keywords:** blood cells, CBC, hematological characteristics, Mendelian randomization, NHANES dataset, stroke risk

## Abstract

**Background and Purpose:**

Stroke remains one of the leading causes of death and disability worldwide, posing a serious global health burden. Identifying reliable and easily accessible biomarkers for stroke risk is therefore of great clinical importance. The complete blood count (CBC) is a routine and widely available hematological test that provides key information about blood cell components, including red blood cells, white blood cells, and platelets. These parameters collectively reflect systemic conditions such as inflammation, infection, and coagulation, which are known to play critical roles in stroke pathogenesis. Recent evidence suggests that certain CBC parameters may be associated with an increased risk of stroke; however, the nature and direction of these associations remain unclear. This study aims to comprehensively evaluate the relationship and potential causal links between individual CBC parameters and the risk of stroke.

**Methods:**

Our analysis encompassed 6736 participants from the NHANES dataset (2011–2016). We utilized a suite of statistical techniques: multivariable logistic regression, Weighted Quantile Sum (WQS) regression, restricted cubic spline analysis, and Mendelian randomization. These methods were applied to assess the link between blood cell metrics (CBC) and stroke, considering a broad spectrum of covariates such as age, race, socioeconomic status, BMI, lifestyle factors (smoking, alcohol consumption, physical activity), serum calcium levels, hypertension, and diabetes. Stroke cases were ascertained using a dedicated questionnaire, and an extensive array of CBC parameters was examined. This study aims to systematically assess the association between CBC parameters and stroke risk, and explore their potential causal relationship using multivariate statistical analysis and genetic epidemiological methods.

**Results:**

Initial analysis revealed significant disparities in age, education, income, hypertension, and diabetes between the stroke and nonstroke cohorts. CBC, notably LBXRDW, LBXHCT, LBXHGB, and LBXMCHSI, emerged as independent predictors of stroke. WQS regression underscored a positive association between these indices and stroke risk, which was attenuated after adjusting for confounders. Restricted cubic spline analysis uncovered significant nonlinear correlations for LBXRDW and LBXMCHSI with stroke events. Mendelian randomization further suggested potential causal links between specific CBC (e.g., mean erythrocyte hemoglobin concentration, red blood cell distribution width) and stroke, albeit with variable correlation directions. Sensitivity analyses, including heterogeneity inspection and pleiotropy testing, reinforced the robustness of these findings.

**Conclusions:**

This study reveals the multi‐layered and complex associations between CBC parameters and stroke risk, emphasizing the need to comprehensively consider hematological characteristics and various confounding factors in stroke‐related research. These findings contribute to a deeper understanding of the mechanisms underlying stroke onset and provide a theoretical basis and new insights for future early screening and personalized interventions for stroke risk.

## Graphical abstract

This study investigated the association and causality between complete blood count (CBC) parameters and stroke in 6736 participants from the 2011–2016 NHANES. Key CBC indices were identified as independent stroke predictors, with some showing significant nonlinear correlations with stroke events. Mendelian randomization revealed potential causal links between multiple blood cell parameters and stroke, with sensitivity analyses confirming result robustness.

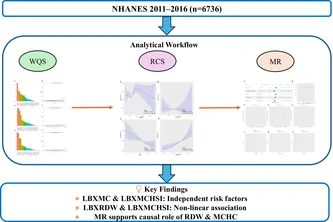

**Abbreviations**
AHA: American Heart Association
BMI: body mass index
CBC: complete blood count
CDC: the Centers for Disease Control and Prevention
LBDBANO: basophils number (1000 cells/μL)
LBDEONO: eosinophils number (1000 cells/μL)
LBDLYMNO: lymphocyte number (1000 cells/μL)
LBDMONO: monocyte number (1000 cells/μL)
LBDNENO: segmented neutrophils num (1000 cells/μL)
LBXBAPCT: basophils percent (%)
LBXEOPCT: eosinophils percent (%)
LBXHCT: hematocrit (%)
LBXHGB: hemoglobin (g/dL)
LBXLYPCT: Lymphocyte percent (%)
LBXMC: MCHC (g/dL)
LBXMCHSI: mean cell hemoglobin (pg)
LBXMCVSI: mean cell volume (fL)
LBXMOPCT: monocyte percent (%)
LBXMPSI: mean platelet volume (fL)
LBXNEPCT: segmented neutrophils percent (%)
LBXPLTSI: platelet count SI (1000 cells/μL)
LBXRBCSI: red blood cell count (million cells/μL)
LBXRDW: red cell distribution width (%)
LBXWBCSI: white blood cell count (1000 cells/μL)
MEC: mobile examination centers
MR: Mendelian Randomization
MRI: magnetic resonance imaging
NCHS: National Center for Health Statistics
NHANES: US National Health and Nutrition Examination Survey
RCS: restricted cubic spline
WQS: weighted quantile sum

## 1 Introduction

Stroke, a predominant causative agent of global morbidity and mortality, represents a multifaceted cerebral impairment triggered by acute cerebrovascular disruptions [1, 2]. Categorically, strokes bifurcate into ischemic, primarily caused by vascular occlusions, and hemorrhagic, resultant of intracranial hemorrhage [3]. Contemporary diagnostic modalities encompass cranial computed tomography (CT), magnetic resonance imaging (MRI), and diverse hematological evaluations, delineating stroke etiology and categorization [4]. Therapeutic interventions diverge based on stroke typology; ischemic variants often necessitate thrombolytic agents for clot dissolution, whereas hemorrhagic instances may require surgical intervention to mitigate bleeding [5]. Notwithstanding, these therapeutic avenues are constrained by limitations such as the time‐sensitive efficacy and hemorrhagic risk of thrombolytic therapy, and the inherent risks and patient‐specific unsuitability of surgical options [6]. Post‐stroke rehabilitation, a critical aspect of recovery, suffers from variable accessibility and efficacy [7]. Hence, advancing research is imperative for enhancing diagnostic precision, extending therapeutic windows, refining treatment modalities, and augmenting rehabilitative methodologies. This encompasses the exploration of novel pharmacotherapies, surgical technique refinement, and the integration of telehealth for optimized stroke management in geographically isolated regions [8].

Beyond conventional risk factors, significant research efforts are directed towards identifying hematological biomarkers for stroke risk stratification, etiology determination, and prognosis prediction [9]. Blood cells and their parameters are readily accessible and routinely measured, positioning them as promising candidates [10]. Current research focuses on several key areas: (1) The role of inflammation, reflected in leukocyte counts (neutrophils, lymphocytes) and derived ratios like the neutrophil‐to‐lymphocyte ratio (NLR), which have been associated with stroke incidence, severity, and outcomes [11]; (2) Platelet indices (mean platelet volume‐MPV, platelet count, platelet distribution width‐PDW) as indicators of platelet activation and hypercoagulability, potentially linked to thrombotic stroke risk [12]; (3)Erythrocyte parameters, including red cell distribution width (RDW), which may signal underlying inflammation or oxidative stress impacting vascular health and stroke susceptibility [13]; (4) The utility of coagulation markers like D‐dimer and fibrinogen, particularly in contexts like embolic stroke of undetermined source (ESUS) or cancer‐associated stroke, where they may indicate occult hypercoagulable states or malignancy [14]. Key findings consistently link elevated NLR, RDW, MPV, and coagulation markers (D‐dimer, fibrinogen) to increased stroke risk and poorer outcomes. However, controversies persist regarding the optimal cutoff values for clinical utility, the independent predictive power beyond established risk factors, and the influence of acute phase reactions post‐stroke on baseline levels [15]. A major research gap lies in establishing robust causal relationships, as observational studies are susceptible to confounding and reverse causality. Furthermore, comprehensive analyses integrating multiple hematological parameters and genetic data to develop predictive models are still evolving [16]. Therefore, there is an urgent need for large‐scale, population‐based investigations that can evaluate both the associative and causal links between CBC parameters and stroke risk. Such work would not only clarify mechanistic pathways connecting systemic inflammation and cerebrovascular injury but also identify novel blood‐based markers for early detection and prevention.

The National Health and Nutrition Examination Survey (NHANES), conducted by the National Center for Health Statistics (NCHS) within the Centers for Disease Control and Prevention (CDC), is instrumental in assessing the health and nutritional status of diverse American cohorts [17]. NHANES intricately synthesizes structured interviews with comprehensive physical examinations, generating a robust dataset crucial for evaluating the American health landscape, particularly in identifying trends and informing policy decisions in cerebrovascular health, such as stroke prevalence [18]. NHANES's in‐depth examination of stroke‐related factors encompasses lifestyle habits, dietary regimes, chronic disease prevalence, and cardiovascular health parameters [19]. This dataset's breadth and depth facilitate an exhaustive analysis of stroke correlations, including gastrointestinal health markers like constipation, mental health conditions such as depression, oral health issues including periodontal disease, adherence to specific diets like the MIND diet, and critical anthropometric measurements like body mass index and waist circumference [20]. It also scrutinizes smoking habits, encompassing both traditional and electronic cigarettes [21]. Critically, NHANES provides extensive hematological data, enabling large‐scale investigation into the associations between blood cell parameters and stroke risk within a representative population.

We aim to determine if blood changes predict stroke risk and identify key blood factors. This research is crucial for understanding these relationships and improving public health. Our two‐part method first examines mediators like income and health factors within NHANES (2011–2016) to understand their link to stroke. Then, we use a two‐step MR approach with genetic variants to establish causality between specific blood parameters and stroke risk, addressing limitations of confounding inherent in observational studies.

We ensure our MR study follows key principles and cross‐references a large meta‐analysis and UK Biobank data for robust results. Our study seeks to reveal how blood factors relate to stroke, potentially finding new risk markers for targeted interventions, with significant implications for stroke research and healthcare.

## 2 Methods

### 2.1 Study Population

The US National Health and Nutrition Examination Survey (NHANES) [22] is a nationwide cross‐sectional survey representative of the noninstitutionalized civilian resident US population. Data were collected from home interviews and standardized mobile examination centers (MEC), which are published in 2‐year cycles. The study procedures were approved by the Ethics Review Board of Health Statistics Research at the First Affiliated Hospital of Nanchang University. Informed consent was obtained from participants before any data collection, and NHANES protocol details are available at the CDC (centers for disease control and prevention; https://www.cdc.gov/nchs/nhanes/index.htm) website. The analytical sample in this study was derived from NHANES 2011–2016 participants 20 years of age or older with complete data on blood cell parameters and covariates (*n* = 6736). As individuals with missing data were excluded, potential selection bias cannot be ruled out, and the findings should be interpreted with caution.

### 2.2 Stroke

The criteria we used in our study were the MCQ (Multiple Choice Questionnaire). Ever told you had a stroke? Questions were used for judgment, in which Yes was judged as yes, No was judged as no, and Refused, Don't know, and missing data were excluded.

### 2.3 Blood Cells

The CBC we used in the study included LBXWBCSI‐White blood cell count (1000 cells/μL), LBXLYPCT‐Lymphocyte percent (%), and LBXWBCSI‐White blood cell count (1000 cells/μL). LBXMOPCT‐Monocyte percent (%), LBXNEPCT‐Segmented neutrophils percent (%), LBXEOPCT‐Eosinophils percent (%), LBXBAPCT‐Basophils percent (%), LBDLYMNO‐Lymphocyte number (1000 cells/μL), LBDMONO‐Monocyte number (1000 cells/μL), LBDNENO‐Segmented neutrophils num (1000 cells/μL), LBDEONO‐Eosinophils number (1000 cells/μL), LBDBANO‐Basophils number (1000 cells/μL), LBXRBCSI‐Red blood cell count (million cells/μL), LBXHGB‐Hemoglobin LBXHGB‐Hemoglobin (g/dL), LBXHCT‐Hematocrit (%), LBXMCVSI‐Mean cell volume (fL), LBXMCHSI‐Mean cell hemoglobin (pg), LBXMC‐MCHC (g/dL), LBXRDW‐Red cell distribution width (%), LBXPLTSI‐Platelet count SI (1000 cells/μL), LBXMPSI‐Mean platelet volume (fL), total 20 variables.

### 2.4 Other Covariates

Age is expressed in years as a continuous variable. Race was categorized into five categories, including Mexican American, other Hispanic, white, black, and other races. Income status was calculated according to the INDFMPIR index, with INDFMPIR < 2 indicating low income, INDFMPIR > 3 indicating high income, and the middle indicating normal level. Education level was defined as a three‐point variable that included less than high school, high school graduate/equivalent, and college or higher. Body mass index (BMI) was expressed as a continuous variable. Smoking status was dichotomized using serum cotinine concentrations above 14 ng/mL indicating a long history of smoking. Drinking status was defined by the question “drinking at least 12 drinks per year”, and participants who answered “yes” were identified as drinkers. Sedentary status was classified according to sedentary time. Serum calcium was expressed as a continuous variable. Hypertension was defined based on 2017 American Heart Association (AHA) recommendations as well as antihypertensive medication use and self‐reported hypertension. Diabetes was diagnosed according to the following criteria: patients with prediabetes or diabetes were defined according to the American Diabetes Association criteria, which were defined as one of the following four conditions: glycated hemoglobin > 5.7% or 6.5%, fasting blood glucose > 100/126 mg/dL, physicist‐diagnosed diabetes or impaired glucose tolerance in the questionnaire [23].

### 2.5 Mendelian Randomization Analysis

We downloaded GWAS summary data on blood cells and stroke from open gwas (https://gwas.mrcieu.ac.uk/) [24]. Screening of SNPS in IV instrumental variables of blood cells. We screened them according to the standard of *p* < 5e‐8 [25]. The parameters to remove linkage disequilibrium were set as *r* ^2^ = 0.001 and kb = 10,000. SNPS with *p* > 5e‐08 in the stroke outcome variable were also excluded after data integration. TwoSampleMR package [26] was used for two‐sample analysis. Heterogeneity and sensitivity analyses were also performed with the use of the TwoSampleMR package, and the IVW (Inverse‐variance weighted) estimates were considered to be causal only if they were statistically significant and did not show evidence of pleiotropy.

### 2.6 Weighted Quantiles and Regressions

WQS regression [24] is a statistical model for multiple regression of high‐dimensional data sets commonly used in environmental exposures. This model allows a weighted index to be constructed in a supervised manner to assess the overall effect of environmental exposure and the contribution of each component of the mixture to the overall effect. We used gWQS package to evaluate blood cells in relation to the overall effect of cerebral apoplexy and combined it with the results of logistic regression analysis.

### 2.7 Restricted Cubic Spline Analysis

We used restricted cubic spline (RCS) analysis to assess whether there was a linear relationship between blood cells and stroke. An important assumption in most regression models is that the independent variables and dependent variables are linearly related, which is difficult to meet in practice. A common solution is to classify continuous variables, but the choice of the number of categories and the location of nodes is often subjective, and classification often loses information. Regression splines are piecewise polynomials requiring continuity and second‐order differentiability at knots, whereas restricted cubic splines additionally impose linearity constraints at both ends of the independent variable's data range [25]. We used the functions in the rms package to perform RCS analysis to evaluate the relationship between the main factors affecting stroke occurrence and stroke occurrence. This study included participants aged 20 and above, in alignment with the design and data availability of the NHANES dataset. The inclusion of younger adults enables the capture of hematological variations and associated stroke risk factors across a wide age range, which is crucial for early detection and prevention. This broad age coverage enhances the generalizability of our findings and aligns with the study's objective to systematically evaluate associations across different age groups.

### 2.8 Statistical Analysis

All data calculations and statistical analyses were performed with the use of R software (https://www.r‐project.org/, version 4.3.1). All continuous variables were expressed as medians (interquartile ranges) and analyzed with the use of the Wilcoxon rank‐sum test, and categorical variables were expressed as frequencies and analyzed with the use of the chi‐square test. For continuous variables related to smoking, such as cotinine concentration, and alcohol consumption, such as total annual alcohol intake, we performed RCS analysis to evaluate their continuous effects across different exposure levels. We used WTMEC2YR for the calculation of weights and the survey package for the calculation of weighted logistic regression and linear regression. All *p* < 0.05 were considered to be statistically significant if they were not specified.

## 3 Results

### 3.1 Baseline Comparison

**FIGURE 1:**
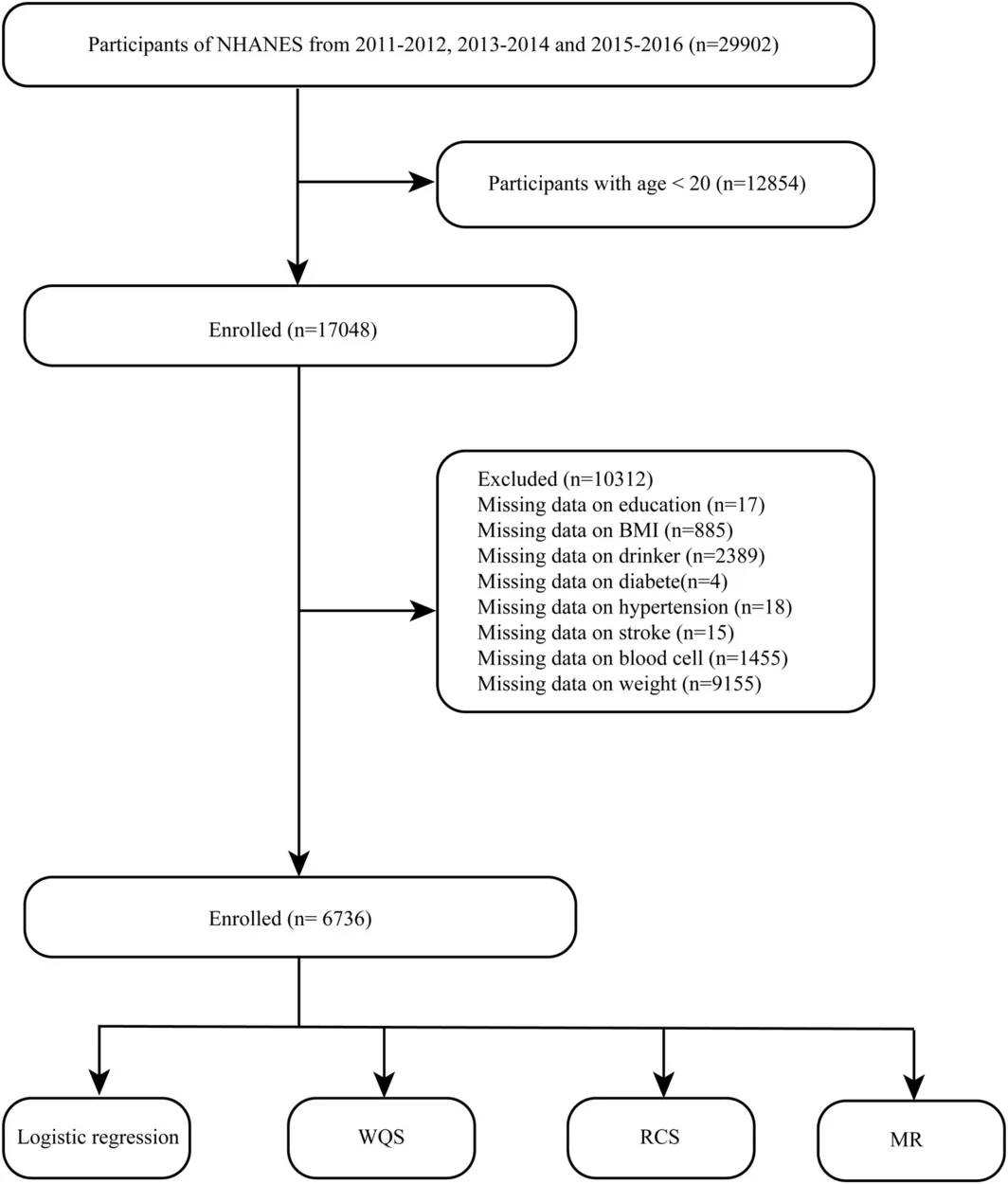
Overall flowchart. BMI, body mass index; MR, Mendelian Randomization; NHANES, National Health and Nutrition Examination Survey; RCS, restricted cubic spline; WQS, Weighted Quantile Sum.

For our analysis, we collected 6736 participants aged 20 years and older from the NHANES database between 2011 and 2016. Details of the screening procedures are provided in Figure 1. The data of 6736 cases was grouped according to whether they had stroke or not. The clinical characteristics and differences of each group are summarized in the baseline data table in Table 1. As can be seen from the Table 1, in the baseline data, there were statistical differences in age, education level, income, whether suffering from hypertension and diabetes between the two groups (*p* < 0.05). Among them, the population in the stroke group was older, with lower education level, higher income, and higher proportion of hypertension and diabetes. There was no significant difference in BMI, race, smoking, and drinking between the two groups (*p* > 0.05). Additionally, we conducted further analyses on continuous variables related to smoking and alcohol consumption. Serum cotinine concentration was used as a continuous indicator for smoking exposure, and total annual alcohol intake was treated as a continuous variable for alcohol consumption. RCS analyses were performed to assess potential nonlinear associations with stroke risk. The results indicated that the associations of both smoking and alcohol consumption with stroke risk remained consistent across exposure levels, with no significant nonlinear relationships detected (all *p* for nonlinearity > 0.05). These findings suggest that including these continuous exposure measures did not change the main conclusions. In addition, in blood cells, LBXRDW, LBXHCT, LBXHGB, LBXRBCSI, LBXWBCSI, LBDEONO, LBDNENO, LBDMONO, LBDLYMNO, LBXBAPCT, LBXEOPCT, LBXNEPCT, a total of 13 indicators of LBXLYPCT had statistical differences between the two groups (*p* < 0.05).

**TABLE 1:** Baseline clinical characteristics and group differences.

Characteristic	Overall, *N* = 6736^a^	Stroke		*p* ^b^
		No, *N* = 6478^a^	Yes, *N* = 258^a^	
LBXMC	33.80 (33.20, 34.40)	33.80 (33.20, 34.40)	33.80 (33.00, 34.40)	0.3
LBXRDW	13.20 (12.70, 13.80)	13.11 (12.70, 13.80)	13.60 (12.90, 14.40)	**< 0.001**
LBXMCHSI	30.50 (29.30, 31.70)	30.50 (29.30, 31.70)	30.65 (29.40, 32.30)	0.3
LBXMCVSI	90.1 (86.9, 93.2)	90.0 (86.9, 93.1)	90.9 (87.3, 95.1)	0.052
LBXHCT	42.3 (39.5, 44.9)	42.3 (39.6, 45.0)	41.2 (38.0, 44.1)	**0.016**
LBXHGB	14.30 (13.40, 15.30)	14.30 (13.40, 15.30)	14.00 (12.70, 15.00)	**0.006**
LBXRBCSI	4.70 (4.39, 5.03)	4.71 (4.40, 5.03)	4.54 (4.16, 4.92)	**0.010**
LBXWBCSI	6.40 (5.50, 7.90)	6.40 (5.50, 7.80)	6.90 (5.60, 8.20)	**0.047**
LBXMPSI	8.30 (7.80, 9.00)	8.30 (7.80, 9.00)	8.40 (7.80, 9.20)	0.2
LBXPLTSI	227 (193, 268)	227 (194, 268)	224 (179, 256)	0.064
LBDBANO				
0	3821 (56%)	3688 (56%)	133 (48%)	
0.1	2815 (43%)	2697 (43%)	118 (50%)	
0.2	76 (1.1%)	70 (1.1%)	6 (2.4%)	
0.3	16 (0.2%)	15 (0.2%)	1 (0.4%)	
0.4	4 (< 0.1%)	4 (< 0.1%)	0 (0%)	
0.5	2 (< 0.1%)	2 (< 0.1%)	0 (0%)	
0.7	1 (0%)	1 (0%)	0 (0%)	
0.8	1 (< 0.1%)	1 (< 0.1%)	0 (0%)	
LBDEONO	0.20 (0.10, 0.30)	0.20 (0.10, 0.30)	0.20 (0.10, 0.30)	**0.004**
LBDNENO	3.70 (2.90, 4.80)	3.70 (2.90, 4.80)	4.10 (3.30, 5.30)	**0.005**
LBDMONO	0.50 (0.40, 0.60)	0.50 (0.40, 0.60)	0.60 (0.47, 0.70)	**0.002**
LBDLYMNO	1.90 (1.60, 2.30)	1.90 (1.60, 2.30)	1.80 (1.30, 2.30)	**0.035**
LBXBAPCT	0.70 (0.50, 1.00)	0.70 (0.50, 1.00)	0.80 (0.60, 1.00)	**0.016**
LBXEOPCT	2.50 (1.70, 3.80)	2.50 (1.70, 3.80)	3.00 (1.90, 4.00)	**0.025**
LBXNEPCT	58 (52, 64)	58 (52, 64)	59 (54, 67)	**0.004**
LBXMOPCT	8.00 (6.60, 9.40)	8.00 (6.60, 9.40)	8.10 (6.71, 9.60)	0.3
LBXLYPCT	30 (25, 35)	30 (25, 35)	28 (21, 32)	**< 0.001**
Hypertension	2580 (34%)	2390 (33%)	190 (71%)	**< 0.001**
Sedentary				
< 3 h	1469 (19%)	1425 (19%)	44 (15%)	0.5
> 6 h	2931 (46%)	2804 (46%)	127 (50%)	
3–6 h	2336 (35%)	2249 (35%)	87 (35%)	
Smoker	1624 (25%)	1544 (24%)	80 (32%)	0.12
BMI	28 (24, 33)	28 (24, 33)	28 (25, 33)	0.7
Income				
High	2229 (45%)	2173 (46%)	56 (31%)	**< 0.001**
Low	3034 (34%)	2884 (34%)	150 (50%)	
Normal	1473 (20%)	1421 (20%)	52 (19%)	
Education				
Above high school	3745 (63%)	3634 (64%)	111 (46%)	**< 0.001**
Below high school	607 (5.0%)	580 (5.0%)	27 (7.2%)	
High school	2384 (32%)	2264 (31%)	120 (47%)	
Race				
Mexican American	885 (8.2%)	863 (8.3%)	22 (4.8%)	0.2
Non‐Hispanic Black	1404 (11%)	1338 (11%)	66 (14%)	
Non‐Hispanic White	2725 (67%)	2596 (67%)	129 (67%)	
Other Hispanic	757 (6.2%)	736 (6.3%)	21 (4.1%)	
Other Race	965 (7.8%)	945 (7.8%)	20 (10%)	
Gender				
Female	3404 (51%)	3272 (51%)	132 (55%)	0.4
Male	3332 (49%)	3206 (49%)	126 (45%)	
Age	47.0 (33.0, 61.0)	47.0 (33.0, 60.0)	66.0 (57.0, 75.0)	**< 0.001**
Diabetes				
Normal	2780 (46%)	2716 (41.9%)	64 (24.8%)	**< 0.001**
Prediabetes	2682 (40%)	2579 (39.8%)	103 (39.9%)	
Diabetes	1274 (14%)	1183 (18.3%)	91 (35.3%)	
Drinker	4807 (77%)	4635 (77%)	172 (75%)	0.5

*Note:* Bold value indicates *p* < 0.05; *p < 0.01; **p < 0.001.

Abbreviations: LBDBANO, Basophils number (1000 cells/μL); LBDEONO, Eosinophils number (1000 cells/μL); LBDLYMNO, Lymphocyte number (1000 cells/μL); LBDMONO, Monocyte number (1000 cells/μL); LBDNENO, Segmented neutrophils num (1000 cells /μL); LBXBAPCT, Basophils percent (%); LBXEOPCT, Eosinophils percent (%); LBXHCT, Hematocrit (%); LBXHGB, Hemoglobin (g/dL); LBXLYPCT, Lymphocyte percent (%); LBXMC, MCHC (g/dL); LBXMCHSI, Mean cell hemoglobin (pg); LBXMCVSI, Mean cell volume (fL); LBXMOPCT, Monocyte percent (%); LBXMPSI, Mean platelet volume (fL); LBXNEPCT, Segmented neutrophils percent (%); LBXPLTSI, Platelet count SI (1000 cells/μL); LBXRBCSI, Red blood cell count (million cells/μL); LBXRDW, Red cell distribution width (%); LBXWBCSI, White blood cell count (1000 cells/μL).

^a^ Median (IQR); *n* (unweighted) (%).

^b^ Wilcoxon rank‐sum test for complex survey samples; chi‐squared test with Rao & Scott's second‐order correction.

### 3.2 The Effect of Blood Cells on Stroke

We first used multivariable logistic regression to assess the effect of blood cell parameters on stroke. To demonstrate robustness, we built three models: Model 1 included only the blood cell variables; Model 2 added stroke‐related covariates (age, education level, income, and hypertension) to Model 1; and Model 3 further adjusted Model 1 for all other covariates (age, race, education level, income, diabetes, hypertension, gender, BMI, drinking status, and smoking status). All results are summarized in Table 2. In Model 1, LBXMC (mean corpuscle hemoglobin concentration), LBXRDW (red blood cell distribution width), and LBXMCHSI (mean corpuscle hemoglobin concentration) emerged as independent predictors of stroke. In Model 2, LBXMC, LBXMCHSI, and LBXMCVSI (mean corpuscular volume) were identified as independent predictors. In Model 3, only LBXMC and LBXMCHSI remained independent predictors. Thus, while LBXMC, LBXRDW, and LBXMCHSI are independent predictors in the unadjusted model, only LBXMC and LBXMCHSI remain consistently predictive in both the partially and fully adjusted models.

**TABLE 2:** Multivariate logistic regression results between blood cell and stroke.

Characteristic	Model 1			Model 2			Model 3		
	OR	95% CI	*p*	OR	95% CI	*p*	OR	95% CI	*p*
LBXMC	0.30	0.10, 0.91	**0.034**	0.19	0.06, 0.64	**0.010**	0.19	0.05, 0.79	**0.026**
LBXRDW	1.20	1.06, 1.37	**0.007**	1.08	0.93, 1.26	0.3	1.05	0.88, 1.25	0.5
LBXMCHSI	4.45	1.07, 18.5	**0.041**	6.42	1.36, 30.3	**0.021**	6.42	1.06, 39.1	**0.045**
LBXMCVSI	0.64	0.38, 1.08	0.091	0.57	0.32, 0.99	**0.046**	0.56	0.29, 1.07	0.074
LBXHCT	1.07	0.44, 2.63	0.9	0.88	0.36, 2.15	0.8	0.89	0.35, 2.26	0.8
LBXHGB	0.69	0.09, 5.34	0.7	1.16	0.15, 9.20	0.9	1.13	0.13, 9.50	0.9
LBXRBCSI	0.90	0.04, 20.2	> 0.9	1.65	0.11, 24.1	0.7	1.47	0.10, 21.6	0.8
LBXWBCSI	0.55	0.01, 23.7	0.7	0.47	0.01, 24.0	0.7	0.40	0.01, 29.6	0.6
LBXMPSI	1.08	0.86, 1.36	0.5	1.17	0.93, 1.46	0.2	1.17	0.91, 1.51	0.2
LBXPLTSI	1.00	0.99, 1.00	0.2	1.00	1.00, 1.00	0.8	1.00	1.00, 1.01	0.8
LBDBANO	0.47	0.01, 20.1	0.7	0.09	0.00, 8.44	0.3	0.07	0.00, 9.59	0.3
LBDEONO	5.47	0.11, 265	0.4	8.17	0.12, 547	0.3	8.62	0.09, 784	0.3
LBDNENO	1.81	0.04, 82.7	0.8	2.13	0.04, 115	0.7	2.44	0.03, 193	0.7
LBDMONO	2.82	0.06, 145	0.6	2.58	0.04, 158	0.6	2.87	0.03, 244	0.6
LBDLYMNO	1.89	0.04, 81.5	0.7	2.18	0.04, 112	0.7	2.59	0.03, 194	0.6
LBXBAPCT	1.37	0.17, 10.8	0.8	1.26	0.14, 11.0	0.8	1.27	0.14, 11.4	0.8
LBXEOPCT	0.82	0.10, 6.76	0.8	0.67	0.08, 5.86	0.7	0.66	0.07, 6.16	0.7
LBXNEPCT	0.83	0.11, 6.46	0.9	0.71	0.09, 5.74	0.7	0.70	0.08, 6.08	0.7
LBXMOPCT	0.83	0.11, 6.46	0.9	0.70	0.09, 5.65	0.7	0.69	0.08, 5.98	0.7
LBXLYPCT	0.80	0.10, 6.20	0.8	0.70	0.09, 5.66	0.7	0.68	0.08, 5.97	0.7

*Note:* Multivariable logistic regression was used to evaluate the association between blood cell parameters and stroke. Model 1 included only blood cell variables; Model 2 additionally adjusted for age, education level, income, and hypertension; Model 3 further adjusted for all remaining covariates (age, race, education level, income, diabetes, hypertension, gender, BMI, drinking status, and smoking status). Independent predictors of stroke were: LBXMC, LBXRDW, and LBXMCHSI in Model 1; LBXMC, LBXMCHSI, and LBXMCVSI in Model 2; and LBXMC and LBXMCHSI in Model 3. Thus, although LBXMC, LBXRDW, and LBXMCHSI were significant in the unadjusted model, only LBXMC and LBXMCHSI remained consistently predictive after partial and full adjustment. Bold value indicates *p* < 0.05; *p < 0.01; **p < 0.001.

Abbreviations: BMI, body mass index; CBC, complete blood count; CI, confidence interval; LBDBANO, basophil number; LBDEONO, eosinophil number; LBDLYMNO, lymphocyte number; LBDMONO, monocyte number; LBDNENO, neutrophil number; LBXBAPCT, basophil percent; LBXEOPCT, eosinophil percent; LBXHCT, hematocrit; LBXHGB, hemoglobin; LBXLYPCT, lymphocyte percent; LBXMC, mean corpuscle hemoglobin concentration; LBXMCHSI, mean corpuscular hemoglobin concentration; LBXMCVSI, mean corpuscular volume; LBXMOPCT, monocyte percent; LBXMPSI, mean platelet volume; LBXNEPCT, neutrophil percent; LBXPLTSI, platelet count; LBXRBCSI, red blood cell count; LBXRDW, red blood cell distribution width; LBXWBCSI, white blood cell count; OR, odds ratio.

### 3.3 WQS Analysis

**FIGURE 2:**
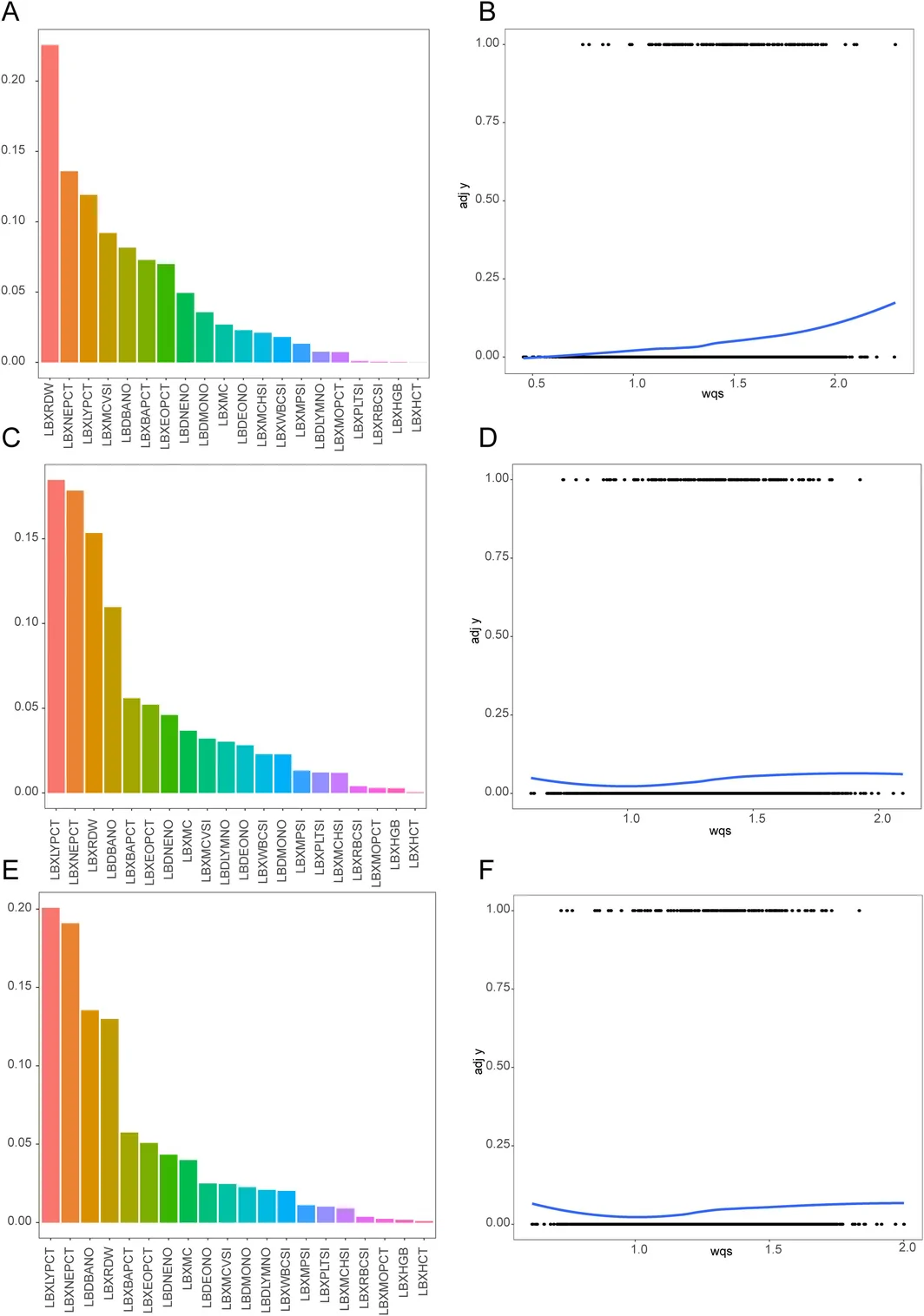
WQS analysis of blood cells for stroke. (A) uncorrected weight of blood cells affecting stroke; (B) unadjusted association between WQS index of blood cells and stroke; (C) partially corrected weight of blood cells affecting stroke; (D) partially corrected for the relationship between blood cell WQS index and stroke; (E) weight of fully corrected blood cells affecting stroke; (F) relationship between fully corrected WQS index of blood cells and stroke. LBDBANO, Basophils number (1000 cells/μL); LBDEONO, Eosinophils number (1000 cells/μL); LBDLYMNO, Lymphocyte number (1000 cells/μL); LBDMONO, Monocyte number (1000 cells/μL); LBDNENO, Segmented neutrophils num (1000 cells/μL); LBXBAPCT, Basophils percent (%); LBXEOPCT, Eosinophils percent (%); LBXHCT, Hematocrit (%); LBXHGB, Hemoglobin (g/dL); LBXLYPCT, Lymphocyte percent (%); LBXMC, MCHC (g/dL); LBXMCHSI, Mean cell hemoglobin (pg); LBXMCVSI, Mean cell volume (fL); LBXMOPCT, Monocyte percent (%); LBXMPSI: Mean platelet volume (fL); LBXNEPCT, Segmented neutrophils percent (%); LBXPLTSI: Platelet count SI (1000 cells/μL); LBXRBCSI: Red blood cell count (million cells/μL); LBXRDW, Red cell distribution width (%); LBXWBCSI, White blood cell count (1000 cells/μL); WQS, Weighted Quantile Sum.

In order to further verify the relationship between global blood cells and stroke and the contribution of each index, we performed WQS analysis. The results without WQS correction showed that there was a positive correlation between CBC and stroke (*p* = 9.08e‐11) (Figure 2B). Figure 2A showed that LBXRDW had the highest contribution. LBXRDW had the highest contribution, followed by LBXNEPCT, LBXLYPCT, LBXMCVSI, LBDBANO, LBXEOPCT and LBXBAPCT; after adjusting for age, education level, income, hypertension, diabetes and other factors, there was no significant correlation between CBC and stroke (*p* = 0.15021, Figure 2D). Figure 2C shows that LBXLYPCT has the highest contribution. The next was LBXNEPCT, LBXRDW, LBDBABO; after adjustment for all, the blood index as a whole and no significant correlation between cerebral apoplexy *p* = 0.42822, Figure 2F, can be seen from the Figure 2E LBXLYPCT contribution is highest, followed by LBXNEPCT, LBDBANO, LBXRDW, LBXBAPCT.

### 3.4 RCS Analysis

**FIGURE 3:**
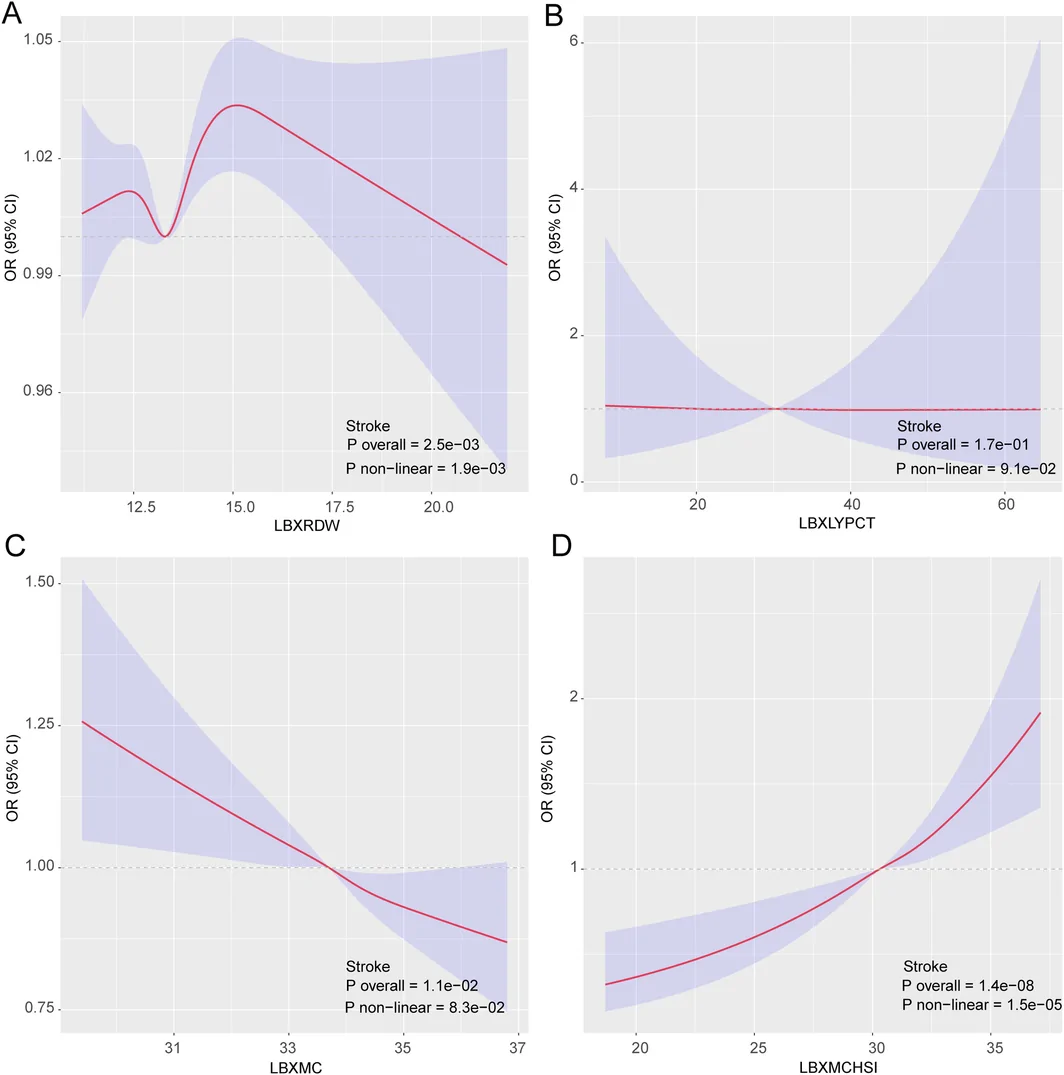
Restricted cubic spline analysis of CBC and stroke. (A) Restricted cubic spline analysis between LBXRDW and stroke; (B) Restricted cubic spline analysis between LBXLYPCT and stroke; (C) Restricted cubic spline analysis between LBXMC and stroke; (D) Restricted cubic spline analysis between LBXMCHSI and stroke. LBXLYPCT, lymphocyte percent (%); LBXMC, mean cell hemoglobin concentration (g/dL); LBXMCHSI: mean cell hemoglobin (pg); LBXRDW, Red cell distribution width (%).

We found from the logistic regression LBXMC, LBXMCHSI effects on cerebral apoplexy is stable. In the WQS analysis, LBXRDW and LBXLYPCT were found to contribute the most, and to further analyze whether there is a nonlinear relationship between these CBC and stroke, restricted cubic spline analysis was performed. It can be seen from the figure that LBXRDW (Figure 3A) has a nonlinear relationship with the occurrence of stroke, and the nonlinear relationship is significant (*p* = 1.9e‐3). There was a nonlinear relationship between LBXMCHSI (Figure 3D) and the occurrence of stroke, and the nonlinear relationship was significant (*p* = 1.5e‐5). There is no nonlinear relationship for LBXLYPCT (Figure 3B) and LBXMC (Figure 3C).

### 3.5 Mendelian Randomization Analysis

Based on the previous analysis, we found that LBXMC, LBXMCHSI, and LBXRDW might be causally related to the development of stroke, and we found GWAS summary data containing these three indicators. From Chen MH et al., a cross‐ethnic meta‐analysis of 15 hematological characteristics in 746,667 participants (including 184,535 individuals of non‐European ancestry) [27], White blood cell count, red blood cell distribution width, red blood cell count, platelet count, neutrophil count, monocyte count, mean platelet volume, mean red blood cell count, platelet count, neutrophil count, monocyte count, mean platelet volume, mean red blood cell volume, mean corpuscular hemoglobin concentration, mean corpuscular hemoglobin, lymphocyte count, hemoglobin concentration, hematocrit, Eosinophil count, basophilic cell count, relevant data could get (Table S1) at https://gwas.mrcieu.ac.uk/. Then the same on the https://gwas.mrcieu.ac.uk/ download ukb‐d‐C_STROKE as the end of the stroke (Table S2).

**FIGURE 4:**
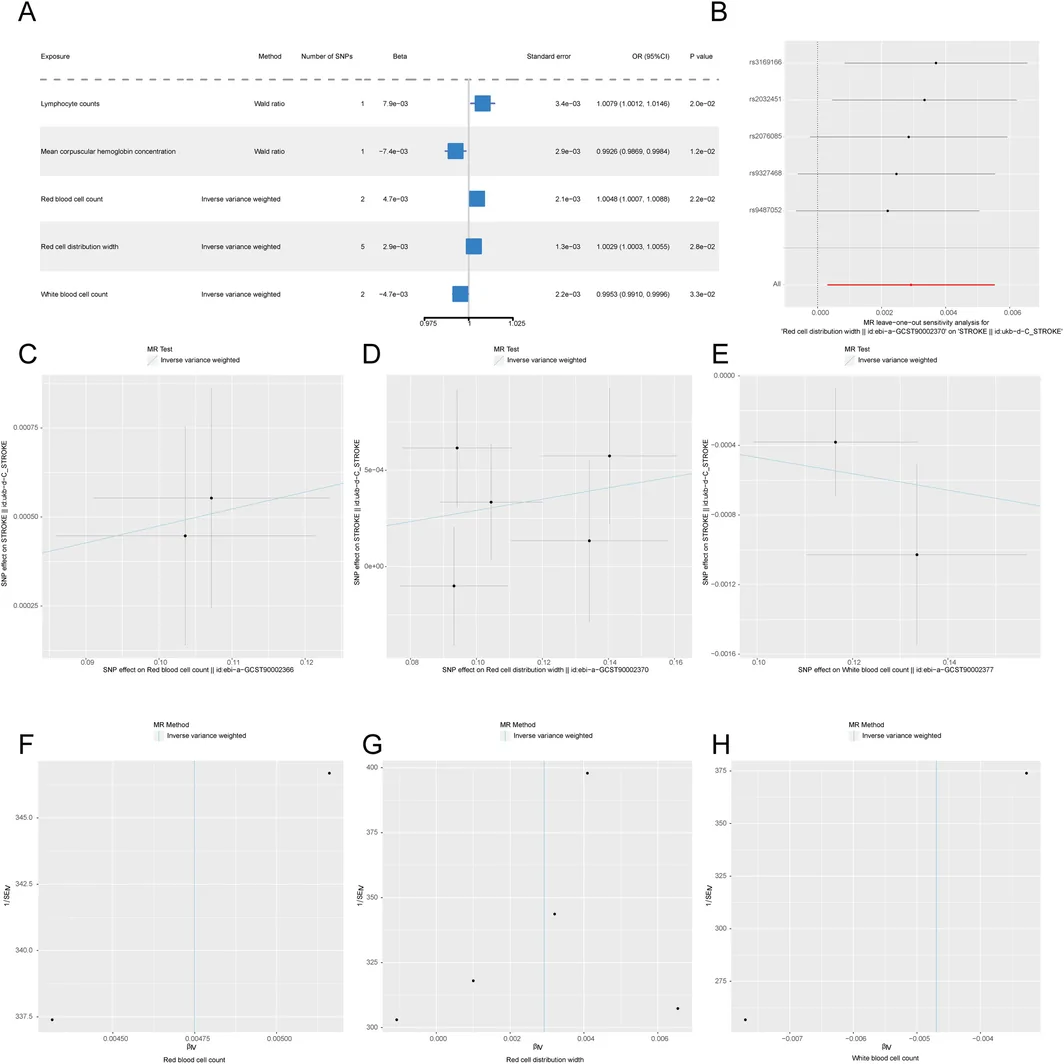
Blood index and Mendelian randomization analysis of cerebral apoplexy. (A) Forest plot of Mendelian randomization analysis results; (B) red blood cell distribution width leave‐one‐out test forest plot; (C) red blood cell count causal analysis of stroke risk scatter diagram; (D) scatter plot of causal analysis of red blood cell distribution width on stroke risk; (E) scatter plot for causal analysis of white blood cell count and stroke risk; (F) funnel plot of effect of red blood cell count on the risk of stroke due to heterogeneity analysis; (G) funnel plot of red blood cell distribution width on stroke risk by heterogeneity analysis; (H) funnel plot of white blood cell count on stroke risk due to heterogeneity analysis.

We examined causality between each of these exposures and outcomes on a case‐by‐case basis with the use of two‐sample Mendelian randomization (Figure 4A; see Table 3 and Table S3 for full results), with the use of the Wald radio method for a number of SNPS of one. Inverse Variance Weighted (IVW) method was used to estimate the causal effect when the number of SNPS was more than 1, and the IVW scatter plot was drawn. The results showed that mean erythrocyte hemoglobin concentration (Wald ratio *p* = 0.1179843) and red blood cell distribution width (Figure 4D, IVW *p* = 0.2827563) had a causal relationship with the occurrence of stroke. In addition, the results of Mendelian randomization showed that red blood cell count (Figure 4C, IVW *p* = 0.2158492), white blood cell count (Figure 4E, IVW *p* = 0.3330559), Lymphocyte count (Wald ratio *p* = 0.1988296) also had a causal relationship with stroke. Among them, mean corpuscle hemoglobin concentration and white blood cell count were negatively correlated with stroke, and the rest were positively correlated with stroke. Because of the small number of SNP results, we performed the steiger direction test, and we found that the *p* values of these three pairs of steiger direction tests were very small, indicating that the occurrence of stroke was caused by the content in the blood (see Table 4).

**TABLE 3:** Mendelian randomization results of blood cell and stroke.

Exposure	Method	nSNP	Beta	SE	*p*	OR	OR (95% CI)
Lymphocyte counts	Wald ratio	1	0.007875906	0.003382323	0.01988296	1.0079070	1.01 [1.00, 1.01]
Mean corpuscular hemoglobin concentration	Wald ratio	1	−0.007421049	0.002947064	0.01179843	0.9926064	0.99 [0.99, 1.00]
Red blood cell count	IVW	2	0.004749452	0.002067142	0.02158492	1.0047607	1.00 [1.00, 1.01]
Red cell distribution width	IVW	5	0.002921194	0.001331786	0.02827563	1.0029255	1.00 [1.00, 1.01]
White blood cell count	IVW	2	−0.004692445	0.002204703	0.03330559	0.9953185	1.00 [0.99, 1.00]

Abbreviations: CI, confidence interval; IVW, Inverse Variance Weighted; OR, odds ratio; SE, standard error; SNP, single nucleotide polymorphism.

**TABLE 4:** Steiger test results of blood cell and stroke.

Exposure	snp_r2.exposure	snp_r2.outcome	correct_causal_direction	steiger_pval
Lymphocyte counts	0.007915353	0.00001544175	TRUE	2.6e‐14
Mean corpuscular hemoglobin concentration	0.008825835	0.00001755519	TRUE	1.1e‐07
Red blood cell count	0.009507580	0.00001473099	TRUE	4.2e‐17
Red cell distribution width	0.021830456	0.00002246942	TRUE	1.7e‐39
White blood cell count	0.009627793	0.00001497174	TRUE	2.7e‐17

Subsequently, we conducted sensitivity analyses to assess the robustness of our findings. We began with an examination of heterogeneity, as detailed in Table 5. The analysis included a funnel mapping of SNP exposures greater than one, focusing on variables such as the average concentration of hemoglobin in red blood cells and the number of SNPs associated with lymphocyte count, where a single SNP was found not to introduce heterogeneity. Furthermore, our evaluation of red blood cell count (as shown in Figure 4F) revealed no significant heterogeneity (*p* = 0.8379531). Similarly, no heterogeneity was detected in the distribution width of red blood cells (Figure 4G, *p* = 0.5081940) and the white blood cell count (Figure 4F, *p* = 0.3488251). Then the pleiotropy test (Table 6) was performed. The pleiotropy test was based on the *p* of the intercept term of egger's method. In this analysis, only SNPS with sufficient red blood cell distribution width were used for pleiotropy test, showing no heterogeneity (*p* = 0.9354143). Finally, the leave‐one‐out test was carried out, and only the red blood cell distribution width had sufficient SNPS for leave‐one‐out forest plot display. Figure 4B showed that the Beta value was relatively stable after eliminating any SNP, indicating that the IVW result of the random model was relatively stable. There is a causal relationship between the two (see Table S4 for details).

**TABLE 5:** Heterogeneity test results of blood metal and stroke.

id.exposure	id.outcome	Outcome	Exposure	Method	*Q*	*Q*_df	*Q*_*p*val
ebi‐a‐GCST90002366	ukb‐d‐C_STROKE	STROKE	Red blood cell count	IVW	0.04182532	1	0.8379531
ebi‐a‐GCST90002370	ukb‐d‐C_STROKE	STROKE	Red cell distribution width	MR Egger	3.29615092	3	0.3481787
ebi‐a‐GCST90002370	ukb‐d‐C_STROKE	STROKE	Red cell distribution width	IVW	3.30466136	4	0.5081940
ebi‐a‐GCST90002377	ukb‐d‐C_STROKE	STROKE	White blood cell count	IVW	0.87772647	1	0.3488251

Abbreviation: IVW, Inverse Variance Weighted.

**TABLE 6:** Pleiotropy test results of blood metal and stroke.

id.exposure	id.outcome	Outcome	Exposure	Egger_intercept	SE	*p*
ebi‐a‐GCST90002370	ukb‐d‐C_STROKE	STROKE	Red cell distribution width	−0.00007933548	0.0009014349	0.9354143

## 4 Discussion

Strokes, major brain events causing neuronal damage, are the second leading cause of death globally, affecting 15 million yearly. In the US 800,000 suffer post‐stroke disabilities annually, costing $46 billion, with rising fatalities highlighting the need for understanding risks for effective prevention and treatment [28, 29].

The NHANES database examines stroke factors like lifestyle, diet, health conditions, and cardiovascular metrics, aiding analysis of stroke links to health and behaviors.

Our study, analyzing NHANES 2011–2016 data of 6736 adults after excluding participants with missing covariates, retained statistically sufficient power for robust inference. Although the exclusion of participants with missing data reduced the initial cohort size, this approach allowed for consistent covariate adjustment and model stability. However, due to the complex and nonuniform patterns of missingness in NHANES, excluded individuals did not constitute a single, comparable group with shared characteristics. Therefore, a direct baseline comparison between included and excluded participants was not feasible. Instead of stating that the final sample remained representative of the NHANES target population, we acknowledge that the exclusion of individuals with incomplete data may introduce potential selection bias, and thus the generalizability of our findings should be interpreted with caution. Sensitivity analyses confirmed the stability of our core findings, supporting the validity of our conclusions despite this limitation. Our multivariate logistic regression analysis, utilizing data from the US HANES, identified specific CBC‐LBXMC, LBXRDW, and LBXMCHSI‐as independent predictors of stroke in unadjusted models. Notably, LBXMC and LBXMCHSI displayed consistent predictive significance in both partially and fully adjusted models, underscoring their potential correlation with stroke incidence across diverse analytical frameworks.

Consistent with extensive epidemiological evidence, our observation that each standard‐deviation increase in RDW is associated with a higher odds of stroke echoes findings from the ARIC and REGARDS cohorts, where elevated RDW conferred 15%–20% excess risk of first‐ever ischemic stroke [30]. In contrast, the inverse association we detected for MCHC adds to the sparse literature—U.S community study has reported a similar protective trend for higher MCHC against hemorrhagic stroke [31], suggesting that improved erythrocyte hemoglobin packing may mitigate cerebrovascular vulnerability. Taken together, these comparisons highlight that RDW primarily captures anisocytosis—often driven by inflammation, oxidative stress, and iron dys‐homeostasis—whereas MCHC may integrate information on intracellular hemoglobin content and oxygen‐carrying efficiency, offering complementary biological insights [13].

Furthermore, our study highlights the consistent role of LBXMC and LBXMCHSI as independent factors in stroke risk across various models. This finding corroborates existing literature on the critical role of anemia and related hematologic abnormalities as indicators of stroke risk [32, 33, 34]. Additionally, we further analyzed the continuous effects of smoking and alcohol consumption using serum cotinine concentrations and total annual alcohol intake as exposure variables, employing RCS analysis. The results showed that the associations of smoking and alcohol consumption with stroke risk remained consistent across different exposure levels, with no significant nonlinear relationships detected. Our WQS regression analysis revealed a positive correlation between overall CBC and stroke risk, with significant contributions from metrics like LBXRDW, LBXLYPCT, and LBXNEPCT. This comprehensive method evaluates the aggregate impact of all CBC, emphasizing the influence of those with higher contributions.

The application of restricted cubic spline analysis in our study uncovered significant nonlinear relationships between LBXRDW, LBXMCHSI, and stroke occurrence, suggesting that the influence of these CBC on stroke risk is not linear but follows a more complex pattern. This complexity may reflect intricate biological mechanisms and interactions with other risk factors or distinct pathophysiological pathways.

Our study, utilizing Mendelian randomization analysis, reveals a causal link between specific CBC and stroke. In this study, we performed Mendelian randomization analysis using GWAS data. Preliminary heterogeneity tests indicated no significant heterogeneity among the included single nucleotide polymorphisms (SNPs). Therefore, we selected the fixed‐effect IVW method for estimating causal effects—a statistically appropriate choice given the absence of significant heterogeneity, as fixed‐effect models provide more accurate and stable estimates under such conditions. The analysis indicates a negative correlation between mean erythrocyte hemoglobin concentration and white blood cell count with stroke, challenging traditional perspectives and inviting further exploration into how changes in blood cell composition might contribute to stroke pathogenesis or prevention. The specific results show that the Wald ratio *p*‐value for mean erythrocyte hemoglobin concentration is 0.1179843, and the IVW *p*‐value for white blood cell count is 0.3330559. Additionally, red blood cell distribution width also shows a causal relationship with stroke, with an IVW *p*‐value of 0.2827563. In contrast, a positive correlation of red blood cell count, lymphocyte count, and red blood cell distribution width with stroke underscores the multifactorial nature of stroke risk. This genetic epidemiological approach minimizes confounding and provides robust evidence for a direct association between these hematological characteristics and stroke.

Our study makes a substantial contribution to the field of stroke research, particularly by highlighting the utility of CBC as biomarkers for stroke risk assessment. It underscores the importance of an integrated approach in elucidating stroke etiology, which combines clinical observations with genetic data. From a translational standpoint, incorporating RDW and MCHC into existing tools such as the Framingham Stroke Risk Profile could enhance early identification of high‐risk individuals, especially in primary‐care settings where advanced imaging or genotyping is unavailable [35]. This comprehensive perspective sets the stage for future investigations aimed at deciphering the complex biological mechanisms that underlie these observed associations. Such research is vital for developing targeted interventions based on the unique characteristics of blood cells.

However, it is imperative to recognize certain limitations of our study. Mendelian randomization, while a powerful tool in reducing confounding influences, is not entirely immune to such factors. First, the use of complete‐case analysis may introduce selection bias due to the exclusion of participants with missing data, which may affect the representativeness of the study population. Second, although widely used, this approach may limit generalizability compared to methods such as multiple imputation. Third, the cross‐sectional design of NHANES precludes definitive conclusions regarding temporal or causal relationships. Furthermore, the cross‐sectional nature of the NHANES dataset presents challenges in establishing definitive temporal and causal relationships. The dependability of our findings is also contingent upon the accuracy and completeness of the NHANES data, which, despite its breadth, may not cover all variables relevant to stroke risk. Another limitation is the inclusion of younger adults (20–40 years), who have a relatively lower incidence of stroke. Their inclusion may have resulted in an underestimation of the associations or introduced age‐related heterogeneity in risk profiles. Future studies should consider conducting age‐stratified analyses, particularly among middle‐aged and older populations, to more accurately characterize stroke risk and refine prevention strategies.

In conclusion, our research significantly advances our understanding of stroke, particularly in the context of CBC as potential indicators of stroke risk. Our findings advocate for a more comprehensive approach to understanding stroke etiology, integrating both clinical and genetic information. Future research should focus on unraveling the detailed biological mechanisms behind these associations and exploring the potential for customized interventions based on blood cell properties. Ultimately, the aim of such research is to refine stroke prevention strategies, thereby reducing the substantial health and economic impacts of this condition on a global scale.

## Author Contributions

Each author has made significant contributions to the conception and development of this work. Specifically, T.C. and Y.L. designed the study, analyzed data, and helped draft the manuscript. L.C. reviewed the literature and contributed to data interpretation. Y.L. conducted experiments. P.K. performed statistical analyses. L.Z. provided valuable insights for the discussion. P.L. supervised the entire study, finalized the manuscript, and served as the corresponding author. All authors approved the final manuscript.

## Funding

The authors have nothing to report.

## Ethics Statement

This study is based entirely on publicly available bioinformatics data. No human participants were directly involved, and no identifiable private information was used. Therefore, institutional review board (IRB) approval and participant consent were not required for this analysis. This study was conducted in accordance with the ethical principles outlined in the Declaration of Helsinki.

## Consent

The authors have nothing to report.

## Conflicts of Interest

The authors declare no conflicts of interest.

## Supporting information


**Table S1:** Hematological characteristics meta‐analysis data.

**Table S2:** Stroke endpoint data from UK Biobank

**Table S3:** Mendelian randomization analysis results.

**Table S4:** Stability of beta values and causal relationship analysis.

## Data Availability

The results supporting the findings of this study are provided in detail in the Tables S1–, S4 accompanying the article, and as such, the raw dataset is not publicly available. This approach ensures transparency of the analyses conducted while protecting the integrity and confidentiality of the original data. For further inquiries regarding the methodologies or results presented, readers are encouraged to contact the corresponding author. We are committed to providing additional analysis details and supporting data within the bounds of privacy protection and data security principles upon reasonable request.
