# Supplementary material for: Mediators of the Association Between Stroke and Blood Cells: A Two‐Step Multivariable Mendelian Randomization Study
Source: CNS Neurosci Ther. 2026 Sep 11;32(9):e70903. doi: 10.1002/cns.70903 (PMC13565345; doi:10.1002/cns.70903)
Supplement: Supplementary file 1 [file CNS-32-e70903-s003.docx]

Supplementary Table 4: Stability of Beta Values and Causal Relationship Analysis

| exposure | outcome | id.exposure | id.outcome | samplesize | SNP | b | se | p |
| --- | --- | --- | --- | --- | --- | --- | --- | --- |
| Lymphocyte counts | STROKE | ebi-a-GCST90002319 | ukb-d-C_STROKE | 361,194 | All |  |  |  |
| Mean corpuscular hemoglobin concentration | STROKE | ebi-a-GCST90002330 | ukb-d-C_STROKE | 361,194 | All |  |  |  |
| Red blood cell count | STROKE | ebi-a-GCST90002366 | ukb-d-C_STROKE | 361,194 | All | 0.004749452 | 0.002067142 | 0.02158492 |
| Red cell distribution width | STROKE | ebi-a-GCST90002370 | ukb-d-C_STROKE | 361,194 | rs2032451 | 0.003341021 | 0.001470147 | 0.02305178 |
| Red cell distribution width | STROKE | ebi-a-GCST90002370 | ukb-d-C_STROKE | 361,194 | rs2076085 | 0.002846090 | 0.001569253 | 0.06973031 |
| Red cell distribution width | STROKE | ebi-a-GCST90002370 | ukb-d-C_STROKE | 361,194 | rs3169166 | 0.003698424 | 0.001455563 | 0.01105710 |
| Red cell distribution width | STROKE | ebi-a-GCST90002370 | ukb-d-C_STROKE | 361,194 | rs9327468 | 0.002463610 | 0.001570954 | 0.11682854 |
| Red cell distribution width | STROKE | ebi-a-GCST90002370 | ukb-d-C_STROKE | 361,194 | rs9487052 | 0.002192392 | 0.001459655 | 0.13309886 |
| Red cell distribution width | STROKE | ebi-a-GCST90002370 | ukb-d-C_STROKE | 361,194 | All | 0.002921194 | 0.001331786 | 0.02827563 |
| White blood cell count | STROKE | ebi-a-GCST90002377 | ukb-d-C_STROKE | 361,194 | All | -0.004692445 | 0.002204703 | 0.03330559 |
